# The Effects of 3D Custom Foot Orthotics with Mechanical Plantar Stimulation in Older Individuals with Cognitive Impairment: A Pilot Study

**DOI:** 10.3390/brainsci12121669

**Published:** 2022-12-04

**Authors:** Lorenzo Brognara, Mayra Alejandra Mafla-España, Isabel Gil-Molina, Yolanda Castillo-Verdejo, Omar Cauli

**Affiliations:** 1 Department of Biomedical and Neuromotor Science, University of Bologna, Via Ugo Foscolo 7, 40123 Bologna, Italy; 2 Frailty and Cognitive Impairment Organized Group, University of Valencia, 46010 Valencia, Spain; 3 Department of Nursing, University of Valencia, Jaume Roig s/n, 46010 Valencia, Spain; 4 Geroresidencais, La Saleta, Colisée Group, 46015 Valencia, Spain

**Keywords:** executive function, cognitive impairment, gait, posture, inertial sensor, fall risk, 3D printing, foot orthosis

## Abstract

Recent scientific evidence supports the idea that foot plantar stimulation increases the functional connectivity of brain regions involved in visuo-spatial and sensory-motor integration. In this before–after, non-randomised intervention study we assessed the change in several gait and postural parameters using inertial sensor measurements after acute plantar stimulation using custom 3D-printed insoles. The pilot study was performed on 22 institutionalised, older individuals with a high comorbidity burden who either walked autonomously or with the help of a cane. The intensity of the effects in the first mechanical plantar stimulation session (at one week) strongly predicted a change in the 180° turn duration (*p* < 0.05) and the standard deviation of the step duration (*p* < 0.05) during the timed up-and-go test. Based on these effects, researchers also predicted decreases in some postural parameters such as the root mean square of displacement on the anterior–posterior axis (*p* < 0.01). Thus, these preliminary findings provide a strong rationale for performing controlled clinical trials with larger samples to investigate the efficacy and mechanisms of mechanical plantar stimulation in frail elderly individuals.

## 1. Introduction

Spatio-temporal gait parameters are considered a clinical characteristic that typifies the prodromal phase of neurocognitive diseases. Indeed, the extensive academic literature suggests that it can be used as an indicator of an individual’s biological age [1,2,3]. Furthermore, recent advances in electronic gait analysis and wearable technology may allow for the more precise estimation of neurocognitive-related changes in motor performance in order to identify older adults who, because of neurocognitive diseases, may have an impaired ability to integrate sensory information to facilitate their performance in terms of postural stability and gait [4].

This article describes a proof-of-concept study designed to assess the potential utility and effectiveness of a new rehabilitation treatment based on mechanical plantar stimulation previously described in the literature in terms of utility and effectiveness [5,6]. The aim of this paper was to examine the effect of mechanical foot plantar stimulation via customised 3D foot orthoses and to verify if these orthoses also promote changes in several spatiotemporal gait and postural parameters in older adults with cognitive decline. In this study, we evaluated different gait and postural parameters (some of which have not been previously investigated) under different conditions; for example, by measuring the motion of the sway of the body while standing with the feet close together, standing still, or after visual perturbation (eyes-open versus eyes-closed). Previous research has shown that considering different parameters (postural and gait), allows researchers to quantify the improvement from a static and dynamic point of view, in fact, the postural parameters provide information about static sway whereas gait parameters provide information on the quality of walking [4,5].

### Cognitive Decline in Older Individuals and Effects of Peripheral ‘Bottom-Up’ Plantar Stimulation

Older adults with impaired or declining gait speed have increased care needs, higher incident disability, shorter survival times, faster cognitive decline, and a higher incidence of dementia [6,7,8,9]. Evidence from neuroimaging studies has confirmed that cognitive functions and gait control share neural networks and genetic determinants, especially in the prefrontal, parietal, and temporal areas [10,11]. In recent years, new rehabilitation strategies have been proposed to provide alternative approaches for gait and postural impairments [12,13,14]. Various studies using different methods have investigated the effects of peripheral ‘bottom-up’ stimulation and the role of sensory receptors such as proprioceptors, in giving continuous feedback to the central nervous system. Considering the sensory deficits presented by patients with neurological impairments, several authors have proposed automated mechanical peripheral stimulation, applied under the foot, as a new and exciting potential rehabilitation treatment [15,16,17,18,19]. Stimulation based on low levels of pressure (0.3–0.9 N/mm^2^) on specific areas of the plantar sole impacts the mechanoreceptors of the feet to generate sensory feedback that is important for perceiving changes to body orientation; this feedback could improve the postural sway and spatiotemporal parameters of gait [5]. Pagnussat et al., found that mechanical plantar stimulation increased resting-state brain connectivity between the right primary sensorimotor areas and the left prefrontal cortex; areas related to sensorimotor information that may be the basis for gait and balance improvements [6].

## 2. Materials and Methods

This was a cross-sectional pilot study performed with individuals with a residential profile who were institutionalised in long-stay centres for the elderly in the province of Valencia between January 2022 and September 2022. The study protocol was approved by the Human Research Ethics Committee at the University of Valencia (Reference number H38417528) and written consent for participation was obtained, after first informing each person and their caregivers in a clear and simple way about the purpose of the study and procedures involved. Individuals were included if they met the following inclusion criteria: (1) aged 60 years or older; and (2) able to walk autonomously or with the help of a cane or walker.

The exclusion criteria were: (1) a history or presence of peripheral sensory neuropathy; (2) any peripheral musculoskeletal conditions that may alter balance and/or gait; (3) lower limb lesions in the past 6 months; (4) history of neurosurgery or orthopaedic surgery; and (5) recent (<3 months) hospitalisation, or a diagnosis of cancer or blindness. Each individual underwent a complete clinical, geriatric, and functional assessment. Seven validated scales were used to evaluate the balance and cognitive areas: the Barthel Index, Tinetti scale, Yesavage scale, a mini-mental test (MEC), Cornell Scale, Norton scale, and Downton fall risk index [20,21,22,23,24,25,26,27].

The Cornell Scale is a 19-item instrument developed specifically to assess depression in older people with dementia. Items are clustered into five categories: cyclic functions, ideational disturbance, mood, behavioural disturbance, and physical signs: a score >10 indicates a probably major depressive episode and a score >18 indicates a major depressive episode [20].

The Norton scale grouped five items: physical condition, mental condition, activity, mobility, and incontinence. For each item, a value of 1 (worst condition) to 4 (best condition) is scored. The sum of the five separate items represents the total Norton score, which varies from 5 to 20. A score <14 indicates a high risk of developing pressure ulcers [21]. The Downton Index assesses items, grouped into 5 categories, which are related to the risk of falls: previous falls, medication, sensory deficit, mental state, and ambulation. A total score greater than or equal to 3 indicates a risk of falls. This is an instrument with high sensitivity to predict fall risk, so its use is very interesting in preventive programs [22]. The Barthel Index assesses the ability to perform the activities of daily life (ADLs), and measures independence with 10 items, with a score range of 0–100. The items assessed are feeding, bathing, grooming, dressing, urine and faecal continence, toilet use, transfers (bed to chair and back), mobility (on level surfaces), and ability to use stairs. A lower score indicates greater dependence, while a higher score indicates greater independence, with 0 representing total dependence and 100 representing total independence. The Cornell Scale assesses the course of the depressive symptoms and consists of 19 items which are rated 0 (no symptoms), 1 (mild symptoms), or 2 (severe symptoms) and also allows a rating “item unable to evaluate” [23]. The Tinetti Scale is one of the most useful tools for assessing the functional level of the population which is composed of the examination of two parts: balance and gait. The gait part has 7 items with a total score of 12 points while in the balance part there are 9 items with a total score of 16 points. The final score of the scale is 28 points and the interpretation is the following: 25–28 = low risk of falls; 19–24 = moderate risk of falls, and <19 = high risk of falls [24]. The Yesavage scale evaluates the depressive symptoms present in the elderly. We used the reduced version, composed of 15 dichotomous response (yes or no) items, with scores ranging from 0 to 15, where a score of over 5 indicates the probable presence of depression. The presence of depression was also dichotomized by reviewing the medical records for a clinical diagnosis of depression (including antidepressant and other psychotropic drug treatments) [25]. The MEC is the Spanish version of the Mini-Mental State Examination (MMSE) and comprises 11 items that screen cognitive impairment by assessing five cognitive areas: orientation (temporal and spatial), attention and calculation, word recall, language, and visuospatial abilities. The maximum MEC score is 35 points, and scores lower than 30 points suggest the presence of cognitive impairment. Specifically, normal cognitive function scores 30–35 points, borderline cognitive deficits score 25–29 points, mild cognitive impairment scores 20–24 points, moderate cognitive impairment scores 15–19 points, and severe cognitive impairment receives ≤14 points [26,27].

Each participant underwent a complete clinical, geriatric, and functional assessment using these scales which are all widely used in the scientific literature to assess cognitive function, dementia, and depression, and to identify people at risk of falling (Table 1). Twenty-two patients were included in this study, 10 women (45%) and 12 men (55%).

After having assessed the general clinical conditions of each patient’s foot, the podiatrist on our team scanned the patient’s feet using a 3D Sense laser scanner (3D Systems, Rock Hill, SC, USA) to obtain a 3D foot scan. These scans were then uploaded into 3D CAD software (Rhinoceros; McNeel, Seattle, WA, USA), which was used to create and generate a 3D model of a custom insole for each patient. These were then printed in a polyurethane-based thermoplastic with a 3D printer using a fused deposition modelling (FDM) technique. We chose this process in order to standardise the insoles and guarantee higher reproducibility compared to the conventional handmade process, as previously demonstrated in a recently published study [28].

**Figure 1:**
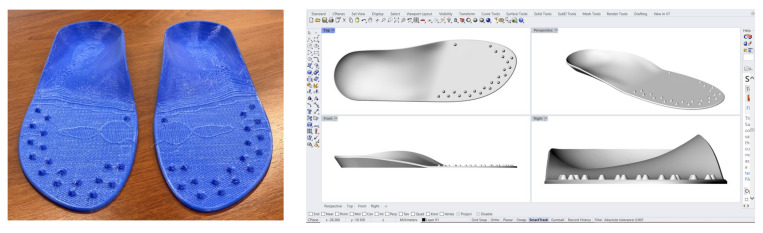
Detail of the 3D printed custom foot orthoses with the blunted cones positioned according to recent reports in the scientific literature [29,30].

The design and shape of the 3D custom foot orthoses (CFOs) with mechanical stimulation were based on recent evidence from the academic literature. In fact, Strzalkowski et al. showed that the distribution of cutaneous nerve afferents on the sole of the foot increases from the medial to lateral aspects of the forefoot [29,30]. The customised 3D-printed insoles we used included various blunted cones (Figure 1) in the plantar area, which have been previously analysed in other published articles.

Every patient wore the 3D custom insoles while the gait and postural analyses were performed at baseline, immediately after treatment (patients walked while wearing the insoles for 30 min), and after 1 week with the participants wearing the 3D custom foot orthotics with peripheral sensory bottom-up stimulation twice a day for 30 min (mid-morning and mid-afternoon). The total experimental period lasted 1 week.

The participants were instructed to walk at their preferred speed at baseline and after plantar stimulation. Gait analysis was conducted using a 7-m timed up-and-go (TUG) test while wearing a portable inertial sensor while wearing their usual comfortable shoes. Furthermore, all the participants were asked to stand still for 30 s (quiet standing) at baseline, immediately after the treatment, and after 1 week to assess their postural control. The trials were performed in four different conditions in the following order: (1) feet open (side by side) with eyes open; (2) feet open (side by side) with eyes closed; (3) feet close together (tandem) with eyes open; and (4) feet close together (tandem) with eyes closed. The portable inertial sensor system was part of the mTest^3^ product range and included the mTUG application for smart devices (mHealth Technologies, Bologna, Italy).

### 2.1. Timed Up-and-Go Test

Associations between physical function and cognitive decline in older adults, as well as the use of the TUG test, have been established to assess functional mobility and status in frail elderly people [30,31]. The participants performed the 7-m TUG test in accordance with recommended guidelines [31,32]. The TUG was performed at baseline, immediately after the use of the CFOs, and after 1 week. Participants were instructed to perform the test “as safely possible”, and no verbal encouragement was provided during testing. In cases where the participant usually used a walking aid (e.g., a walker or cane), this aid was also used during testing. The TUG test was performed as follows: at the signal to begin the test (the predefined signal was a ringing noise), the participant stood up from a chair, walked to the marker 7 m away, turned around, and returned to the chair. The test ended when the participants remained seated and still for 3 consecutive seconds. The application then gave the audio signal to conclude the test and the test automatically ended; the report for the selected test was displayed and the predefined parameters (Table 2) were recorded.

### 2.2. Postural Stability Tests

To analyse the postural stability test we used a reliable and validated IMUs wearable posturographic sensor system (mSway, mHealth Technologies, Bologna, Italy). Participants were instructed to finish four standing tasks: standing with their feet open in a comfortable position for 30 s, standing with their feet open but eyes closed for 30 s, with their feet together (tandem) and eyes open for 30 s, and finally, with their feet together (tandem) and eyes closed for 30 s. These postures and periods were chosen in accordance with specific tasks defined by Lord et al. [33]. Postural/balance assessment was analysed by measuring the body sway motion (see Table 3) while standing still with the participant’s feet open or closed, with or without visual perturbation (eyes open or closed). Patients autonomously held the position, and the assessment was always conducted in safe conditions with the presence of practitioners nearby, especially in the test where their eyes were closed and their feet close together. Patients were instructed to hold the position for 30 s until the application sounded the audio signal that the test would automatically end; the report and data for the selected tests were then exported for statistical analysis (Table 3).

### 2.3. Statistical Analysis

The quantitative variables were subjected to a descriptive analysis using central tendency and dispersion measures (mean and standard deviation from the mean). Likewise, a descriptive analysis was also performed for the qualitative variables based on the frequency distributions. Kolmogorov–Smirnov tests were used to estimate the normal distribution of quantitative variables and thus, to define the type of statistical analysis test to use (parametric or nonparametric). If the data sets were non-normally distributed, the data were tested for significance using the Wilcoxon signed-rank test (2 related samples) and Friedman’s test (>2 related samples). The correlation between quantitative variables was evaluated using nonparametric Spearman correlations. A 95% confidence level with a statistical significance of *p* < 0.05 was used for all the analyses and SPSS software (version 26.0, IBM Corp., Armonk, NY, USA) was used for all the statistical analyses.

## 3. Results

### 3.1. Characteristics of the Study Sample

Data collection (socio and clinical variables, geriatric assessments’ scales, and data for sensors analyses) were tabulated in an SPSS file and statistically analysed to observe any association between the main outcomes of the study before the intervention. Then pre and post comparisons in the data obtained with of the sensors’ parameters were analysed to observe which parameters improve after the intervention.

Most of the participants were autonomous in terms of their general mobility. In the activities of daily living, 41% were independent (100 points on the Barthel scale), while 59% were mildly dependent (>60 points). Scores on the MMSE indicated that 41% had normal cognitive function (30–35 points); 36% had mild cognitive impairment (20–24 points); 18% had moderate impairment (15–19 points), and there was severe impairment in 5% (0–14 points). The Yesavage scale identified that 67% of the participants had no symptoms of depression (0–5 points); 24% had mild depression (6–9 points), and 10% had substantial symptoms of depression. The Norton scale scores showed that 73% had a low risk of pressure ulcers (>14 points) while 27% had a medium risk (13–14 points). The Tinetti scale, which evaluated the gait and balance of the participants, identified 41% as having a low risk of falling; 32% had a medium risk, and 27% had a high risk of falling. Finally, the Downton scale scores indicated that 73% were at a high risk of falling (>3 points); 9% were at medium risk (1–2 points), and 18% were at a low risk (0–1 points).

### 3.2. Changes in Gait, Posture, and Geriatric Evaluation Parameters

**Figure 2:**
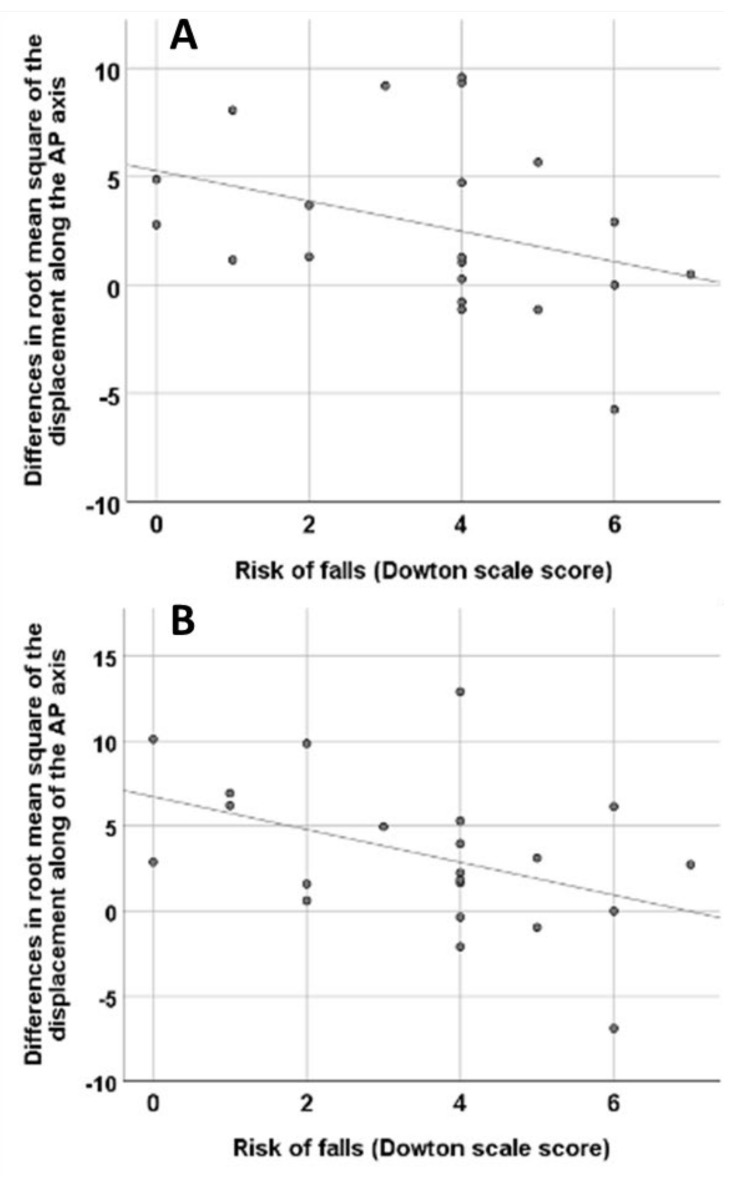
(**A**): Correlation between the differences between the T1 (after 30 min wearing the insoles) and T0 (baseline) tandem root mean square of displacement on the AP axis (*p* = 0.048, Spearman correlation), and the Downton scale. (**B**): Correlation between the differences between the T2 (after one week wearing the insoles) and T0 tandem root mean square of displacement on the AP axis (*p* = 0.047, Spearman correlation) and the Downton scale score.

The variables that appeared to be significantly different after treatment, as shown in Table 3, were further analysed by calculating the difference between each of them at times T1 and T2 by subtracting the baseline value. We assessed these differences to see if there was a significant association with the comprehensive geriatric assessment data. Spearman correlation showed that there was a significant association between these variables, T1 OARoot, tandem mean square of displacement on the AP axis, T0 tandem EO RMS of displacement on the AP axis (*p* = 0.048), T2 tandem EO RMS of displacement on the AP axis, T0 tandem EO RMS of displacement on the AP axis (*p* = 0.047), and the Downton scale (Figure 2). However, no significant association was found between the Downton scale and T1 180° turn duration−T0 180° turn duration (*p* = 0.343), T2 180° turn duration−T0 180° turn duration (*p* = 0.346), T1 standard deviation of step duration−T0 standard deviation of step duration (*p* = 0. 546), T2 standard deviation of step duration−T0 standard deviation of step duration (*p* = 0.131), T1 tandem EO sway path horizontal plane−T0 tandem EO sway path horizontal plane (*p* = 0.360), T2 tandem EO sway path horizontal plane−T0 tandem EO sway path horizontal plane (*p* = 0.582) variables.

### 3.3. The Effect of Mechanical Plantar Stimulation Orthotic Therapy on the Postural Stability Test Results

**Figure 3:**
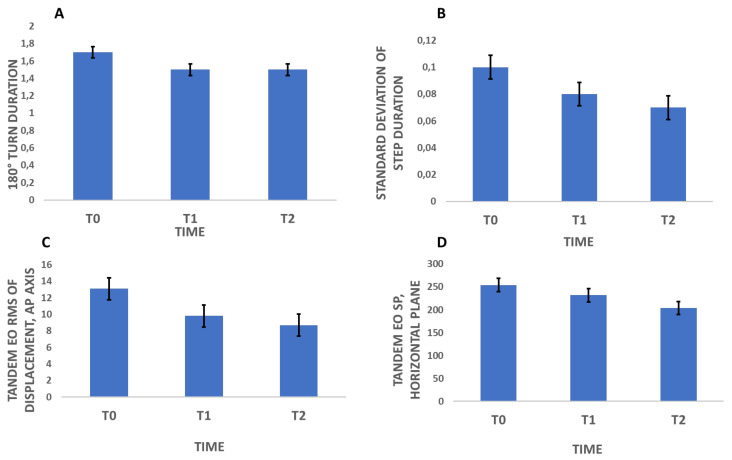
Mean Values of the variables that significantly changed after plantar stimulation (180° turn duration (**A**), Standard deviation of step duration (**B**), Tandem EO RMS of displacement, AP axis (**C**) and Tandem EO SP, horizontal plane (**D**)).

In the postural stability test, custom foot orthotics with mechanical plantar stimulation orthotic therapy significantly lowered the mean oscillations measured in the first use and produced a stable improvement after 1 week for some parameters (the sway path horizontal plane [*p* = 0.041] (Figure 3) and root mean square of displacement on the anterior-posterior axis [*p* = 0.005] (Figure 3), with the eyes open and feet close together in both cases). Many values showed significant trends that will be important to consider in future studies with larger samples. In particular, the mean sway velocity on the anterior-posterior axis (with eyes open) and sway area (with eyes open and feet close together) both showed significant differences (*p* = 0.058). Finally, the sway path on the medio-lateral axis (with eyes open and feet close together) also presented an interesting change (*p* = 0.080) as shown in the Supplementary Table S1.

### 3.4. The Effect of Mechanical Plantar Stimulation Orthotic Therapy on the Timed Up-and-Go Test Results

In the TUG test, custom foot orthotics with mechanical plantar stimulation orthotic therapy significantly lowered the duration of the 180-degree turn performed after having travelled the indicated distance (180° turn duration; *p* = 0.048) as well as the standard deviation of the step duration (*p* = 0.017), as shown in Figure 3. Gait speed also improved from the first use, with a stable improvement after 1 week (*p* = 0.074), although future studies with a larger sample size may be useful to investigate the effect of custom foot orthotics with mechanical plantar orthotic stimulation on gait speed in more depth.

## 4. Discussion

The integration of spatio-temporal gait parameters into clinical assessments of individuals with and without neurocognitive impairment can be easily implemented in the clinical setting via simple, non-invasive, and inexpensive metrics [34,35]. Patients with an amnestic mild cognitive impairment show lower gait speed, which is associated with reduced grey matter volume and cortical thickness; they also show higher stride length variability which is correlated with reduced grey matter volume in spread regions and a thinner cortex in the middle right frontal gyrus [11]. Gait and postural instability are major motor symptoms in individuals with neurocognitive disease and are one of the independent risk factors for falling. Early evidence reported an association between poor performance on physical function measures and worsening cognitive function and supported the use of rehabilitation therapy in improving strength, step length, balance, mobility, and walking endurance.

Physical function and cognitive function share common neurological processes and contribute to cognitive decline, and in tandem with the loss of muscle strength and function, place elderly people at increased risk of personal injury, poor mobility, and fall-related injury experienced during standing and walking, thereby leading to frailty, decreased independence, and poorer quality of life [36,37,38,39,40]. Thus, non-pharmacological treatments such as the use of insoles with sensory stimulation can be a useful rehabilitation strategy that can be integrated into their therapeutic plans. This new bottom-up rehabilitation therapeutic approach using 3D-printed insoles to study gait control in older people is an innovative method based on preliminary studies carried out by our team [5]. This work sheds new light on this exciting field of research from a multidisciplinary perspective and reflects the interplay between geriatrics, neurological and psychiatric sciences, and other health sciences at the leading edge of this field. This work provides new opportunities for improving care or preventing adverse outcomes in several disorders or clinical situations.

Our results demonstrated that 3D-printed CFOs with mechanical plantar stimulation changed kinematic parameters such as the standard deviation of the step duration and 180° turn duration during the TUG test in patients with mild cognitive decline. The present study compared gait spatio-temporal parameters and postural parameters before and after mechanical peripheral stimulation in frail elderly patients with cognitive decline and demonstrated an effect on some of these outcomes. In particular, mechanical plantar stimulation improved some parameters considered variables useful in the assessment of the walking stability and rhythmicity correlated with fall risk and faster cognitive deterioration [41,42].

Our results suggest that the use of 3D custom foot orthotics with mechanical plantar stimulation in older individuals with cognitive impairment strongly predicted a decrease in the root mean square of displacement on the AP axis (*p* = 0.05) in a postural test performed with the feet together (tandem) and eyes open. Furthermore, this study produced results that corroborate the findings of previous work in this field. Hsieh et al. were the first to determine that a smartphone can discriminate between older adults at a lower or higher risk of falling and, in particular, that the root mean square of displacement AP axis appears to distinguish between levels of fall risk during semi-tandem and tandem stance tests [43]. Our protocol and results with foot plantar stimulation agree with the results obtained by other reports; in fact, findings by Pizzigalli et al., show that some parameters, including the anterior-posterior axis sway, discriminate older adult fallers from non-fallers [44]. Based on the results obtained, it is possible to deduce that mechanical plantar stimulation through 3D printed insoles improve some gait and postural parameters and share the same neural effects reported in the literature by some authors that, thanks to a functional magnetic resonance imaging studies, have demonstrated that mechanical plantar stimulation increased resting-state brain connectivity between the right primary sensorimotor areas and the left prefrontal cortex; areas related to sensorimotor information that may be the basis for a gait and balance improvements [6,19].

In our study, the Spearman correlation showed a significant association between the root mean square of displacement on the AP axis and the Downton scale. Nonetheless, despite all the above, this is a new research frontier and good quality trials will still be needed to study the effects of foot orthoses on improving gait and posture in patients with cognitive decline with a view to preventing falls and improving dual-tasking ability. We are aware of the limited size of this present study as its main limitation. However, in our opinion, our sample was of sufficient size for a pilot study in order to create future research directions in this interesting field. Adequately powered randomised clinical trials that also integrate neuroimaging studies to characterise the effects on brain activity will be required to rigorously test this hypothesis. Only some parameters recorded with the sensors were significantly changed after the intervention suggesting that longer wearing time or different locations of sensors in the foot plantar may be tested in future studies in order to achieve strong results in gait parameters. However, the novelty of this intervention, even in this pilot study, opens new directions for this research field in individuals with cognitive impairment in order to reduce the risk of falls and related-adverse outcomes.

### Limitations and Future Direction

Studies with larger samples, which include in their methods, different levels of cognitive impairments, are still needed to better investigate the benefits of foot orthoses on postural analysis and TUG tests. Furthermore, an analysis also on individuals who conduct a sedentary life draw more reliable conclusions.

This study is not without limitations: one limitation of the present study concerns the evaluation of the effects of foot orthoses on postural balance and TUG tests, the long-term effects have been evaluated at week one. For these reasons, further studies should evaluate the effects at one year in order to better understand the effects over time and the long-term clinical impact of this new approach.

## Figures and Tables

**Table 1:** Mean ± standard error mean (SEM) of the age and results for the scales used for geriatric evaluation.

Variable	Mean SEM	Min.–Max.
Age	75.8 ± 1.5	(50–95)
Functional status (Barthel score)	79.7 ± 2.9	(30–100)
Cognitive functions (MMSE test)	23.0 ± 1.2	(0–35)
Depressive symptoms (Yesavage score)	2.8 ± 0.5	(0–12)
Depressive symptoms in dementia (Cornell scale)	3.2 ± 1.4	(0–20)
Pressure ulcer risk assessment (Norton scale)	17.5 ± 0.3	(13–20)
Gait and balance (Tinetti scale)	21.7 ± 0.947	(0–28)
Fall risk assessment (Downton scale)	4.22 ± 0.304	(0–10)

**Table 2:** Description of the spatiotemporal parameters of the timed up-and-go test analysed during pre- and post-mechanical peripheral stimulation.

Name [Unit of Measurement]	Description
Total duration [s]	Total duration of the test. This value is the standard output of the timed up-and-go test. Using mTUG application, this value was automatically measured by the signal recorded by the wearable sensor using a validated algorithm. In the traditional timed up-and-go test this value is measured by healthcare professionals using a stopwatch.
180° turn duration [s]	Duration of the 180-degree turn performed by participants after having travelled the indicated distance.
Sitting turn duration [s]	Duration of the turn performed by the participant in order to sit.
Total number of steps	Total number of steps performed during the timed up-and-go test.
Mean step length [m]	Mean step length (ratio of the travelled distance to the number of steps).
Gait speed [m/s]	Gait speed (ratio of the distance travelled to the time elapsed during the walking phase).
Number of steps in the 180° turn	Number of steps during the 180-degree turn.
Standard deviation of the step duration [s]	Standard deviation of the step duration.
Total duration, starting with the initial chair rise [s]	Total duration of the test starting from the moment the participant rises from the seat.
Sit-to-walk duration [s]	Duration of the initial phase: from the moment the participant rises from the seat to the moment they are upright and begin to walk.

**Table 3:** Postural parameters analysed during the pre- and post-mechanical peripheral stimulation.

Name [Unit of Measurement]	Description
Sway path of the displacement along the anterior–posterior (AP) axis [mm]	Length of the sway path travelled from the centre of mass (CoM) during the oscillation in the AP axis.
Sway path of the displacement along the medio-lateral (ML) axis [mm]	Length of the sway path travelled from the CoM during the oscillation in the ML axis.
Sway path of the displacement on the horizontal plane [mm]	Length of the sway path travelled from the CoM during the oscillation in the horizontal plane. The horizontal plane was defined as the combination of the AP and ML axes.
Sway area [mm^2^/s]	Area travelled by the CoM per second.
95% confidence interval of the ellipse area [mm^2^]	Confidence ellipse area containing 95% of the trajectory points on the horizontal plane (AP and ML exes).
Mean sway velocity along the AP axis [mm/s]	CoM mean sway velocity along the AP axis.
Mean sway velocity along the ML axis [mm/s]	CoM mean sway velocity along the ML axis.
Root mean square of the displacement along the AP axis [mm]	Root mean square value of the displacement (with respect to the centre) along the AP axis.
Root mean square of the displacement along the ML axis [mm]	Root mean square value of the displacement (with respect to the centre) along the ML axis.

## Data Availability

Data will be available upon specific request to the corresponding author.

## Supplementary Materials

The following supporting information can be downloaded at: www.mdpi.com/article/10.3390/brainsci12121669/s1, Table S1: Comparisons between the dynamic (times up-and-go, TUG, test) and static (postural tests) parameters before and after the use of 3D custom foot orthotics (CFOs) with mechanical plantar stimulation, comparing the moment without the CFOs at the baseline (T0) with the first use (immediately after therapy, after wearing the insoles for 30 minutes; T1), and medium-term use (after one week of therapy wearing the insoles; T2) measurements.
