# Supplementary material for: The Effects of 3D Custom Foot Orthotics with Mechanical Plantar Stimulation in Older Individuals with Cognitive Impairment: A Pilot Study
Source: Brain Sci. 2022 Dec 4;12(12):1669. doi: 10.3390/brainsci12121669 (PMC9775314; doi:10.3390/brainsci12121669)
Supplement: Supplementary file 1 [file brainsci-12-01669-s001.zip › brainsci-2071807-supplementary.pdf]

Supplementary Table S1. Comparisons between the dynamic (times up-and-go, TUG, test) and static (postural tests) parameters before and after the use of 3D custom foot orthotics (CFOs) with mechanical plantar stimulation, comparing the moment without the CFOs at the baseline (T0) with the first use (immediately after therapy, after wearing the insoles for 30 minutes; T1), and medium-term use (after one week of therapy wearing the insoles; T2) measurements.

Variable	Chi-square	Degree of difference	*P*-value (Friedman test)	Mean	Standard deviation	Min.	Max.
T0 total duration	4.45	2	.108	30.5	10.7	17.2	60.1
T1 total duration				30.3	11.7	17.3	59.1
T2 total duration				27.8	9.7	15.3	48.4
T0 sit-to-walk duration	0.09	2	.956	1.3	.21	.8	1.9
T1 sit-to-walk duration				1.2	.2	.7	2.0
T2 sit-to-walk duration				1.3	.3	.8	2.3
T0 180° turn duration	**6.09**	**2**	**.048**	4.2	1.7	2.0	8.1
T1 180° turn duration				3.8	1.5	1.9	6.9
T2 180° turn duration				3.6	1.5	1.8	8.0
T0 sitting turn duration	0.85	2	.654	3.0	1.5	1.3	7.1
T1 sitting turn duration				3.1	1.7	1.2	8.0
T2 sitting turn duration				2.9	1.4	1.1	6.6
T0 total number of steps	1.56	2	.458	36.1	14.9	20	87
T1 total number of steps				37.7	18.5	20	94
T2 total number of steps				34.8	14.9	19	73
T0 mean step length	1.30	2	.522	.5	.1	.1	.7
T1 mean step length				.5	.1	.1	.8
T2 mean step length				.5	.2	.1	.8
T0 gait speed	5.21	2	.074	.7	.2	.3	1.2
T1 gait speed				.8	.3	.3	1.4
T2 gait speed				.9	.3	.1	1.6
T0 number of steps in 180° turn	1.23	2	.538	6.4	3.2	2	13
T1 number of steps in 180° turn				6.1	3.3	2	14
T2 number of steps in 180° turn				6.1	3.1	3	15

T0 standard deviation of step duration	**8.13**	**2**	**.017**	.1	.06	.05	.3
T1 standard deviation of step duration				.08	.02	.05	.1
T2 standard deviation of step duration				.07	.02	.03	.1
T0 total duration, starting with the initial chair rise	3.54	2	.170	29.7	10.8	16.7	60.1
T1 total duration, starting with the initial chair rise				29.5	11.7	16.5	58.3
T2 total duration, starting with the initial chair rise				27.0	9.7	14.6	47.1
T0 EO RMS of displacement, AP axis	0.70	2	.705	10.9	6.5	3.7	28.8
T1 EO RMS of displacement, AP axis				10.8	11.4	2.0	52.9
T2 EO RMS of displacement, AP axis				9.9	5.6	2.8	20.7
T0 EO RMS of displacement, ML axis	2.10	2	.350	3.1	3.1	.6	14.5
T1 EO RMS of displacement, ML axis				3.3	2.6	.3	10.2
T2 EO RMS of displacement, ML axis				2.3	1.3	.9	5.5
T0 EO SP, AP axis	3.70	2	.157	184.	75.1	88.5	355.5
T1 EO SP, AP axis				200.0	129.0	69.4	558.8
T2 EO SP, AP axis				162.6	64.7	62.9	326.5
T0 EO SP, AP axis and ML axis	1.90	2	.387	65.8	49.2	26.2	256.1
T1 EO SP, AP axis and ML axis				73.0	51.6	24.8	235.5
T2 EO SP, AP axis and ML axis				59.9	25.1	32.7	152.1
T0 EO SP, horizontal plane	1.90	2	.387	208.8	91.1	96.7	479.7
T1 EO SP, horizontal plane				228.1	143.8	78.6	587.3
T2 EO SP, horizontal plane				184.1	69.5	80.8	381.9
T0 EO SA	0.70	2	.705	17.8	29.1	2.1	136.0

T1 EO SA				22.9	37.0	.7	133.4
T2 EO SA				11.7	11.5	2.8	53.7
T0 EO RMS of displacement, AP axis	10.80	2	.705	10.9	6.5	3.7	28.8
T1 EO RMS of displacement, AP axis				10.8	11.4	2.0	52.9
T2 EO RMS of displacement, AP axis				9.9	5.6	2.8	20.7
T0 EO RMS of displacement, ML axis	0.53	2	.350	3.1	3.1	.6	14.5
T1 EO RMS of displacement, ML axis				3.3	2.6	.3	10.2
T2 EO RMS of displacement, ML axis				2.3	1.3	.9	5.5
T0 EO SP, AP axis	2.10	2	.157	184.9	75.1	88.5	355.5
T1 EO SP, AP axis				200.0	129.0	69.4	558.8
T2 EO SP, AP axis				162.6	64.7	62.9	326.5
T0 EO SP, ML axis	2.50	2	.387	65.8	49.2	26.2	256.1
T1 EO SP, ML axis				73.0	51.6	24.8	235.5
T2 EO SP, ML axis				59.9	25.1	32.7	152.1
T0 EO SP, horizontal plane	6.40	2	.387	208.8	91.1	96.7	479.7
T1 EO SP, horizontal plane				228.1	143.8	78.6	587.3
T2 EO SP, horizontal plane				184.1	69.5	80.8	381.9
T0 EO SA	5.70	2	.705	17.8	29.1	2.1	136.0
T1 EO SA				22.9	37.0	.7	133.4
T2 EO SA				11.7	11.5	2.8	53.7
T0 EO mean SV, AP axis	0.90	2	.058	5.1	2.1	1.9	9.6
T1 EO mean SV, AP axis				5.0	2.6	1.9	13.5
T2 EO mean SV, AP axis				4.1	1.3	1.8	7.1
T0 EO mean SV, ML axis	2.30	2	.368	1.5	.63	.72	2.97
T1 EO mean SV, ML axis				1.6	.7	.6	3.7
T2 EO mean SV, ML axis				1.5	.4	.8	2.4

T0 tandem EO RMS of displacement, AP axis	**4.30**	**2**	**.005**	13.1	8.0	5.1	39.0
T1 tandem EO RMS of displacement, AP axis				9.8	6.8	2.8	29.9
T2 tandem EO RMS of displacement, AP axis				8.7	4.9	2.8	24.3
T0 tandem EO RMS of displacement, ML axis	0.32	2	.767	5.8	6.5	1.2	31.2
T1 tandem EO RMS of displacement, ML axis				5.5	4.1	2.3	19.5
T2 tandem EO RMS of displacement, ML axis				4.4	2.3	1.3	9.2
T0 tandem EO SP, AP axis	1.30	2	.350	189.9	98.4	78.0	455.2
T1 tandem EO SP, AP axis				173.1	90.2	76.9	401.1
T2 tandem EO SP, AP axis				150.9	80.3	50.6	344.8
T0 tandem EO SP, ML axis	0.10	2	.287	129.4	90.3	43.9	462.2
T1 tandem EO SP, ML axis				117.4	61.5	35.1	291.9
T2 tandem EO SP, ML axis				105.6	58.1	38.6	311.0
T0 tandem EO SP, horizontal plane	0.30	2	.041	254.0	135.0	99.6	667.6
T1 tandem EO SP, horizontal plane				232.0	114.6	90.9	531.3
T2 tandem EO SP, horizontal plane				203.9	104.3	71.7	523.4
T0 tandem EO SA	1.30	2	.058	37.4	61.7	5.7	272.2
T1 tandem EO SA				28.7	39.3	3.5	165.3
T2 tandem EO SA				22.7	37.9	1.9	179.4
T0 tandem EO mean SV, AP axis	0.10	2	.638	4.8	2.2	1.7	11.7
T1 tandem EO mean SV, AP axis				4.5	2.2	1.5	10.2
T2 tandem EO mean SV, AP axis				4.0	2.0	1.1	8.2
T0 tandem EO mean SV, ML axis	0.70	2	.316	3.2	1.7	1.2	8.4
T1 tandem EO mean SV, ML axis				3.0	1.4	1.0	5.7
T2 tandem EO mean SV, ML axis				2.7	1.2	1.0	5.8
T0 EC RMS of displacement, AP axis	0.30	2	.116	8.4	3.5	3.4	16.4
T1 EC RMS of displacement, AP axis				11.4	8.0	1.9	34.7

T2 EC RMS of displacement, AP axis				8.8	7.6	2.2	37.6
T0 EC RMS of displacement, ML axis	0.32	2	.848	2.1	1.3	.4	5.5
T1 EC RMS of displacement, ML axis				2.8	2.6	.5	10.1
T2 EC RMS of displacement, ML axis				2.4	2.4	1.0	12.0
T0 EC SP, AP axis	1.30	2	.522	190.2	64.7	68.2	315.3
T1 EC SP, AP axis				207.7	119.3	70.0	503.7
T2 EC SP, AP axis				198.9	126.2	65.1	652.9
T0 EC SP, ML axis	0.10	2	.951	54.7	22.0	26.4	106.1
T1 EC SP, ML axis				73.8	82.3	25.3	408.7
T2 EC SP, ML axis				68.7	63.4	32.4	330.9
T0 EC SP, horizontal plane	0.30	2	.861	207.6	68.9	84.0	351.1
T1 EC SP, horizontal plane				236.9	154.5	85.0	734.8
T2 EC SP, horizontal plane				224.7	155.6	79.9	817.6
T0 EC SP area	3.10	2	.212	10.0	7.8	1.9	33.2
T1 EC SP area				38.8	112.3	1.6	510.4
T2 EC SP area				25.5	77.6	2.0	354.2
T0 EC mean SV, AP axis	0.10	2	.951	4.9	1.7	1.7	8.8
T1 EC mean SV, AP axis				5.3	2.3	1.7	11.3
T2 EC mean SV, AP axis				4.9	2.0	1.9	8.6
T0 EC mean SV, ML axis	0.70	2	.705	1.4	.5	.7	2.6
T1 EC mean SV, ML axis				1.5	.6	.7	3.1
T2 EC mean SV, ML axis				1.5	.5	.9	2.8
T0 tandem EC RMS of displacement, AP axis	0.73	2	.692	10.4	5.9	3.4	27.7
T1 tandem EC RMS of displacement, AP axis				8.9	5.5	2.7	23.0
T2 tandem EC RMS of displacement, AP axis				8.6	4.6	1.2	17.9
T0 tandem EC RMS of displacement, ML axis	0.42	2	.810	4.9	2.8	.9	11.7

T1 tandem EC RMS of displacement, ML axis				4.5	3.1	1.0	12.3
T2 tandem EC RMS of displacement, ML axis				4.0	3.1	1.4	16.2
T0 tandem EC SP, AP axis	2.21	2	.331	214.1	85.7	74.6	447.5
T1 tandem EC SP, AP axis				207.5	121.6	47.8	528.3
T2 tandem EC SP, AP axis				193.0	106.4	50.6	427.0
T0 tandem EC SP, ML axis	5.05	2	.080	147.4	73.9	44.7	282.5
T1 tandem EC SP, ML axis				127.6	78.4	26.7	318.0
T2 tandem EC SP, ML axis				108.7	53.5	30.4	254.3
T0 tandem EC SP, horizontal plane	1.68	2	.431	288.0	118.0	94.7	586.3
T1 tandem EC SP, horizontal plane				268.0	151.2	58.8	629.5
T2 tandem EC SP, horizontal plane				226.7	97.7	64.5	470.2
T0 tandem EC SP area	4.52	2	.104	31.0	35.7	2.2	158.8
T1 tandem EC SP area				28.7	39.1	1.6	154.5
T2 tandem EC SP area				16.1	10.7	2.7	45.9
T0 tandem EC mean SV, AP axis	3.89	2	.143	5.7	2.2	1.9	12.1
T1 tandem EC mean SV, AP axis				5.7	3.4	1.3	15.3
T2 tandem EC mean SV, AP axis				4.7	2.5	1.2	11.9
T0 tandem EC mean SV, ML axis	2.84	2	.241	3.9	1.9	1.2	7.2
T1 tandem EC mean SV, ML axis				3.3	1.9	.6	7.2
T2 tandem EC mean SV, ML axis				2.9	1.4	.6	7.0

Abbreviations used in this table: AP = anterior-posterior; ML = medio-lateral; RMS = root mean square; SV = sway velocity; SPA = sway path area; SP = sway path; EO = eyes open; EC = eyes closed; SA = sway area; tandem = with the feet together.
